# Eye-Opening Approach to Norovirus Surveillance

**DOI:** 10.3201/eid1608.100093

**Published:** 2010-08

**Authors:** Anette Hulth, Yvonne Andersson, Kjell-Olof Hedlund, Mikael Andersson

**Affiliations:** Swedish Institute for Infectious Disease Control, Solna, Sweden

**Keywords:** Viruses, surveillance, norovirus, enteric infections, food-borne infections, vomit, database searches, Sweden, letter

**To the Editor**: It is said that a picture is worth a thousand words. The Figure illustrates this axiom and provides several new insights into the spread of norovirus infections. These infections are assumed to greatly affect society, but little is known about the prevalence of the disease in the community. Samples sent to laboratories usually originate from hospitalized persons and thus give a good view of the situation in healthcare settings. We suspect, however, that these numbers do not depict the true prevalence of norovirus infections in society. We therefore present a new approach to estimate the number of cases and spread of norovirus infections in the community.

We plotted the number of queries for *vomit* (asterisks denote any prefix or suffix) submitted to the search engine on a medical website in Sweden (www.vardguiden.se). This number was normalized to account for the increasing use of the website over time and aggregated by week, starting with week 40 in 2005. We also plotted the number of norovirus findings per week from 16 regional laboratories, as recorded by the Swedish Institute for Infectious Disease Control.

For the time series on Web search queries and laboratory findings (Figure), we fitted harmonic functions on the half-year with no or little activity, defining baselines for each series (*1**,**2*). By performing this procedure, we can identify the onset of each activity that is assumed to occur when the level rises above the 99% prediction interval of the baseline. The week this increase occurs is shown in the Figure. The Figure also contains the number of media articles on winter vomiting disease provided by a search engine for news in Sweden (www.eniro.se/nyhetssok). By analyzing the figure and investigating the statistical outcomes, we glimpse the prevalence of norovirus infections in society, as estimated by the search pattern.

**Figure:**
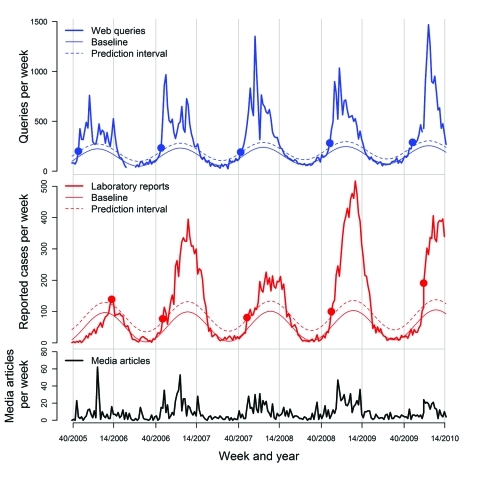
Number of queries for *vomit* submitted to a medical Web site (A), number of laboratory-verified norovirus samples (B), with baselines and 99% prediction intervals, and number of media articles about winter vomiting disease (C) in Sweden, 2005–2010.

We found 3 striking insights. First, the onset of vomiting in the community precedes the onset of confirmed norovirus infections in healthcare settings. In 3 of the 4 full seasons investigated, this precedence was 1–4 weeks. Second, the curve for the Web queries shows much sharper increases and decreases than does the curve on the number of reported norovirus findings. Third, neither search behavior nor reporting of positive tests is driven by media for the winter vomiting disease (confirmed by a linear regression).

In the 2005–06 season, the laboratory reporting raised above the defined prediction interval in week 13, much later than the Web queries. This season had no new variants of norovirus genotype GII.4. This season still showed community infections, even though few reports came from institutions. For the current season (2009–10), the interval between onset of Web queries and onset of norovirus infections in hospitals (week 46 and week 1, respectively) was 8 weeks. In comparison with previous seasons, this delay could mean a low total number of reported cases. However, in late December, a new variant of GII.4 affected healthcare settings in southern Sweden with increasing norovirus infections, while the rest of the country still showed relatively low virus activity.

Other pathogens such as rotavirus, *Salmonella* spp., *Staphylococcus aureus*, and *Bacillus cereus* can cause vomiting. Usually in Sweden, rotavirus infections peak in late winter, and bacterial diseases have a minor incidence compared with norovirus. In our opinion, these other pathogens would not interfere with the interpretation of the results.

In our routine surveillance of Web queries, we also include other query terms, such as diarrhea and stomach flu. However, searches for vomiting show the most distinct pattern, and vomiting is the most pronounced symptom of a norovirus infection.

The use of harmonic functions for describing baseline Web searches and laboratory reporting is a simple model, especially because the parameters are estimated by using the half-year with the least activity. Nonetheless, it is a direct approach, and we believe that the method still captures the time of onsets well.

Web queries indicate the presence of norovirus infections in communities. Predictions of the onset of the norovirus laboratory reporting should also be possible, but further studies are needed to confirm that theory. Web queries have previously been correlated with influenza (*3**–**7*) and have been explored retrospectively for listeriosis (*8*), *Salmonella* spp (*9*), West Nile virus, and respiratory syncytial virus (*10*). With the Web queries, we get an additional surveillance system for the time of the year when few norovirus tests are conducted. In addition, knowing more about the impact of norovirus in the community means that we could provide more adequate information and advocate wiser measures for prevention and control.
